# Global absolute quantification reveals tight regulation of protein expression in single *Xenopus* eggs

**DOI:** 10.1093/nar/gku661

**Published:** 2014-07-23

**Authors:** Arne H. Smits, Rik G.H. Lindeboom, Matteo Perino, Simon J. van Heeringen, Gert Jan C. Veenstra, Michiel Vermeulen

**Affiliations:** 1 Department of Molecular Biology, Faculty of Science, Radboud Institute for Molecular Life Sciences, Radboud University Nijmegen, Nijmegen, The Netherlands; 2 Cancer Genomics Netherlands, Faculty of Science, Radboud Institute for Molecular Life Sciences, Radboud University Nijmegen, Nijmegen, The Netherlands; 3 Department of Developmental Molecular Biology, Faculty of Science, Radboud Institute for Molecular Life Sciences, Radboud University Nijmegen, Nijmegen, The Netherlands

## Abstract

While recent developments in genomic sequencing technology have enabled comprehensive transcriptome analyses of single cells, single cell proteomics has thus far been restricted to targeted studies. Here, we perform global absolute protein quantification of fertilized *Xenopus laevis* eggs using mass spectrometry-based proteomics, quantifying over 5800 proteins in the largest single cell proteome characterized to date. Absolute protein amounts in single eggs are highly consistent, thus indicating a tight regulation of global protein abundance. Protein copy numbers in single eggs range from tens of thousands to ten trillion copies per cell. Comparison between the single-cell proteome and transcriptome reveal poor expression correlation. Finally, we identify 439 proteins that significantly change in abundance during early embryogenesis. Downregulated proteins include ribosomal proteins and upregulated proteins include basal transcription factors, among others. Many of these proteins do not show regulation at the transcript level. Altogether, our data reveal that the transcriptome is a poor indicator of the proteome and that protein levels are tightly controlled in *X. laevis* eggs.

## INTRODUCTION

Recent technological developments have enabled single cell studies at the level of genomics, transcriptomics, proteomics and metabolomics (1,2). For example, next generation sequencing technology allows unbiased and comprehensive sequencing of RNA from single cells (3–5). These pioneering studies have revealed cellular heterogeneity and stochastic gene expression at the single-cell level, which are overseen in analyses on cell populations. Such single cell variations can be caused by differences in cell-cycle phase, developmental stage, local signaling concentrations, transcriptional bursting or genetic alterations.

The first single-cell proteomics studies were performed using fluorescent reporter proteins. These studies all reported noise in the expression of the reporters (6–8). In a more recent pioneering study, a library of yeast strains containing over 2500 endogenously green fluorescent protein (GFP)-tagged proteins (9) was used to measure single-cell protein abundance of these genes. This study revealed that stochastic protein expression upon environmental changes is specific to particular gene classes (10). However, generating comprehensive libraries of endogenously tagged proteins is very labor intensive and impractical for higher eukaryotes. Recently, mass cytometry emerged as a novel technology to study the absolute abundance of proteins in single cells without the need to tag proteins but using endogenous antibodies to which heavy metals are coupled. This method currently allows quantifying up to 32 cellular proteins at the single-cell level in a single experiment (11,12). During the last decade, mass spectrometry-based proteomics emerged as a powerful tool to study the cellular proteome in an unbiased and comprehensive manner (13,14). The current state-of-the-art allows characterizing proteomes to substantial depth from as little as 10.000 mammalian somatic cells. Comprehensive mass spectrometry-based analyses of single mammalian cells is not yet feasible with current instrumentation and methods. Certain cells types, however, contain much more protein. Examples include oocytes or eggs which are several orders of magnitude larger than somatic cells.

For many years, *Xenopus embryos* have been used to study early vertebrate development, elucidating key principles of gene regulation, cellular signaling, patterning and morphogenesis (15). Sequencing and assembly of the *Xenopus tropicalis* genome revealed a very good synteny with the human genome and has allowed characterizing chromatin state and transcription factor binding associated with the maternal to zygotic transition, mesoderm induction and gastrulation (16–19). It also allowed to uncover embryonic transcriptome dynamics and identify patterns of transcript adenylation and deadenylation (20–22). Due to the less complete genome annotation of *Xenopus laevis*, genome-wide transcriptome analyses have been more difficult (23,24). Coincidently, single *X. laevis* eggs contain enough protein for global mass spectrometry-based proteomic analyses.

Developmental processes are known to be tightly controlled, but it is currently unknown to what extent stochastic gene expression plays a role from the beginning of embryogenesis. Here, we present a comprehensive absolute proteome of multiple fertilized *X. laevis* eggs and compare it to both single egg transcriptomes and single gastrula stage embryonic proteomes. The single egg and single gastrula proteomes are both tightly controlled, thus revealing a reduced role for stochastic effects at the beginning of vertebrate development. Developmental dynamics at the protein level are not reflected well by changes at the mRNA level, thus emphasizing the importance of using mass spectrometry-based proteomics to study early embryogenesis.

## MATERIALS AND METHODS

### Animal procedures

*X. laevis* embryos were obtained by *in vitro* fertilization of eggs from a single female, collected at the appropriate stage and snap-frozen in liquid nitrogen. Turned, uncleaved eggs were collected 60 min post-fertilization and gastrulating embryos were staged according to Nieuwkoop and Faber (25) and collected at stage 10.5. Briefly, 12–14 h before egg collections *X. laevis* females were injected with 700 u of human choriogonadotropin (Brevactid 1500 I.E., Ferring) and eggs were fertilized *in vitro*. Embryos were de-jellied in 2% cysteine hydrochloride (Sigma) pH8 in 0.25X measles, mumps and rubella. Fertilized eggs (stage 1 embryo, 60 min post-fertilization) and embryos (gastrula stage 10.5) were collected in an independent tube, snap-frozen in liquid nitrogen and stored at −80°C until further processing for protein or RNA isolation.

### Whole cell extract

Five fertilized egg and five embryos were used for proteome analysis. Each egg and embryo was thawed and homogenized in 15 μl ice-cold low-salt whole cell extract (WCE-LS) buffer (20 mM Tris-HCl pH8, 70 mM KCl, 1 mM ethylenediaminetetraacetic acid (EDTA), 10% glycerol, 5 mM DTT, 0.125% Nonidet P-40, 1 mM phenylmethylsulfonyl fluoride (PMSF), 1x complete EDTA-free protease inhibitor (Roche)). Homogenates were then centrifuged in an Eppendorf tabletop centrifuge at 4°C for 2 min at maximal speed. Supernatant was harvested, snap-frozen and stored at −80°C until mass spectrometry analysis.

### RNA isolation and sequencing

Five fertilized egg and five embryos were used for RNA sequencing. Samples from the same stage were processed in parallel to minimize technical variability. Total RNA was isolated using TRizol® Reagent (Ambion) and RNeasy Kit (Quiagen), and rRNA was depleted with Ribo-Zero™ rRNA Removal Kits (Epicentre). rRNA-depleted RNA was then heat-fragmented in 40 mM Tris-acetate, 100 mM potassium acetate and 30 mM magnesium acetate (pH8.2) and used for cDNA synthesis. First, cDNA strand was obtained from random examers primers using Superscript III (Invitrogen) followed by second strand synthesis using *Escherichia coli* DNA polymerase I (NEB), *E. coli* DNA ligase (NEB) and T4 DNA polymerase (Promega). Purified cDNA was used for strand-specific Illumina sample preparation. Briefly, cDNA was ligated with adapter sequences, size selected (300 bp) and amplified by polymerase chain reaction (PCR). Libraries were sequenced on HiSeq2000 (Illumina). For embryo 10.5 samples, purified cDNA was used for strand-specific Illumina sample preparation using Kapa Hyper Prep Kit, according to manufacturer protocol. To confer strand-specificity a USER enzyme (NEB) digestion step was performed prior to library amplification. Quality control for rRNA depletion, library fragment size and pre-sequencing PCR amplification linearity were carried out on a C1000 thermal cycler coupled with CFX96 Real-Time System reader (BioRad) using iQ SYBR Green Supermix (BioRad) and on an Agilent Bioanalyzer.

### RNA sequencing data analysis

The RNA-Seq experiments resulted in 42 bp single-end sequences that were mapped to the *X. laevis* genome (JGI 7.1) using gmap (26), allowing one mismatch and were stored in BAM files. BamTools API 1.0.2 (27) was used to sort and index the reads. Transcript abundance was calculated by quantifying the Reads Per Kilobase of transcript per Million reads mapped (RPKM value) using Cufflinks v2.1.1 (28). To compare the transcriptomes of the *X. laevis* eggs and embryos 10.5 with high confidence (Figure 4C and Supplementary Figure S3A), the RNA-Seq profiles were merged for each developmental stage using the BamTools API, followed by the removal of random sequences with SAMtools v0.1.18 (29) until the amount of mapped reads were similar.

**Figure 1.**
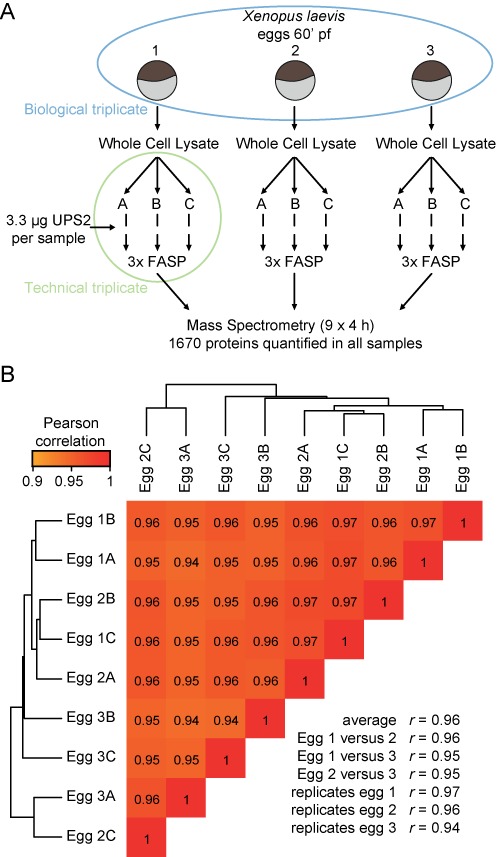
Tight regulation of the single-cell proteome in *X. laevis* eggs. (A) Overview of the workflow used to detect technical and biological variation in single cell proteomics. (B) Unsupervised correlation-based clustering of global proteomes mixes technical and biological replicates. See also Supplementary Tables S2 and S3.

**Figure 2.**
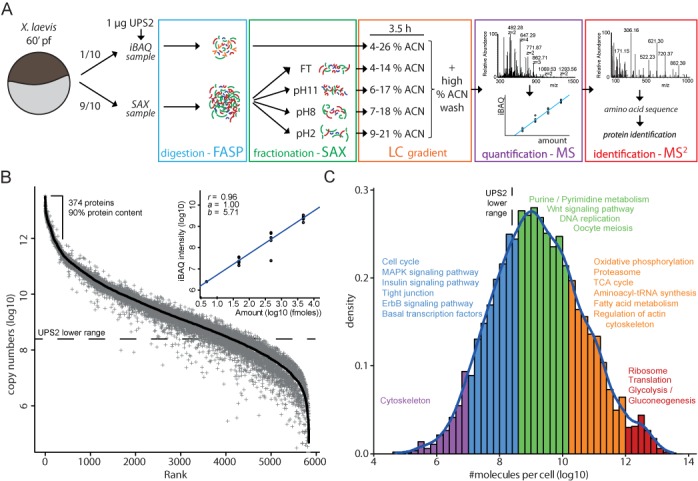
Absolute, deep proteome of individual *X. laevis* eggs. (A) Schematic representation of the workflow used to obtain an absolute, deep proteome. Both the number of fractions and their corresponding nanoLC gradients were optimized for the low amount of protein obtained from a single *X. leavis* egg. (B) The absolute abundance of all proteins in *X. leavis* eggs spans over seven orders of magnitude. The black line corresponds to the mean abundance and the gray plusses represent the standard deviation (five replicates). The dashed line indicates the lowest detected protein of the UPS2 spike-in (unique peptides >2) above which abundance can be accurately determined. The inset represents a typical linear regression curve of the measured iBAQ intensities and the known amounts of UPS2 standard. (C) Distribution of the protein copy numbers in *Xenopus* eggs. Arbitrary cut-offs define five abundance regions for which significant enriched GO terms (FDR < 0.0005) are plotted above these regions. Note the shoulder of high abundant proteins on the right side of the distribution. See also Supplementary Tables S2 and S4.

**Figure 3.**
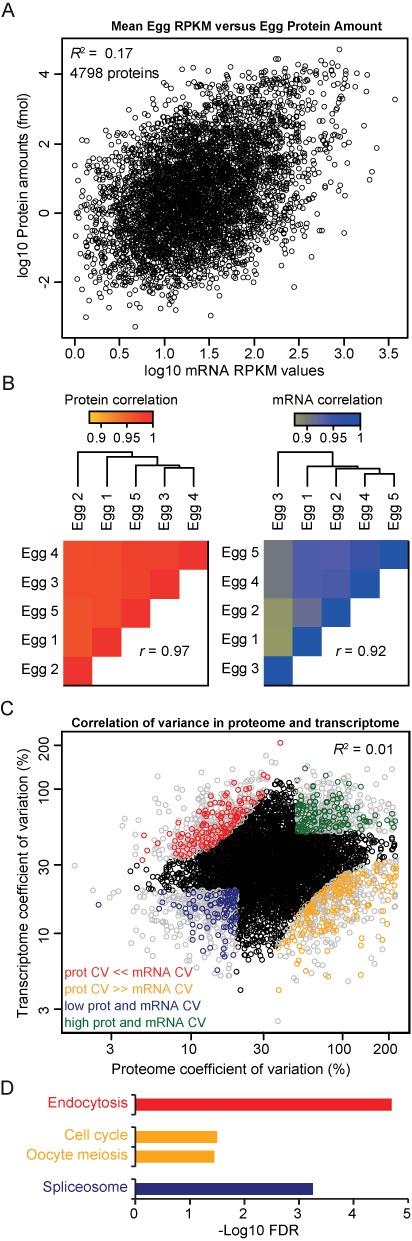
Comparison of single-cell proteomes and transcriptomes. (A) Scatterplot of the mean protein and mRNA levels for genes quantified in all individual proteomes and transcriptomes (4798 genes) reveals a poor correlation between protein and mRNA abundance. (B) Unsupervised correlation-based clustering of global proteomes (left panel) and transcriptomes (right panel) indicate tight regulation of global proteome and transcriptome. (C) Scatterplot of protein and mRNA CV (4798 genes) reveals a lack of correlation between protein and mRNA variation. Genes are colored that show similar mRNA and protein variation (green, high variation; blue, low variation) and that show differential mRNA and protein variation (red, low protein and high mRNA variation; orange, high protein and low mRNA variation). Genes outside these categories are in black. (D) Enriched GO terms for the gene groups as depicted in C. *Y*-axis represents the –log FDR. See also Supplementary Table S4.

**Figure 4.**
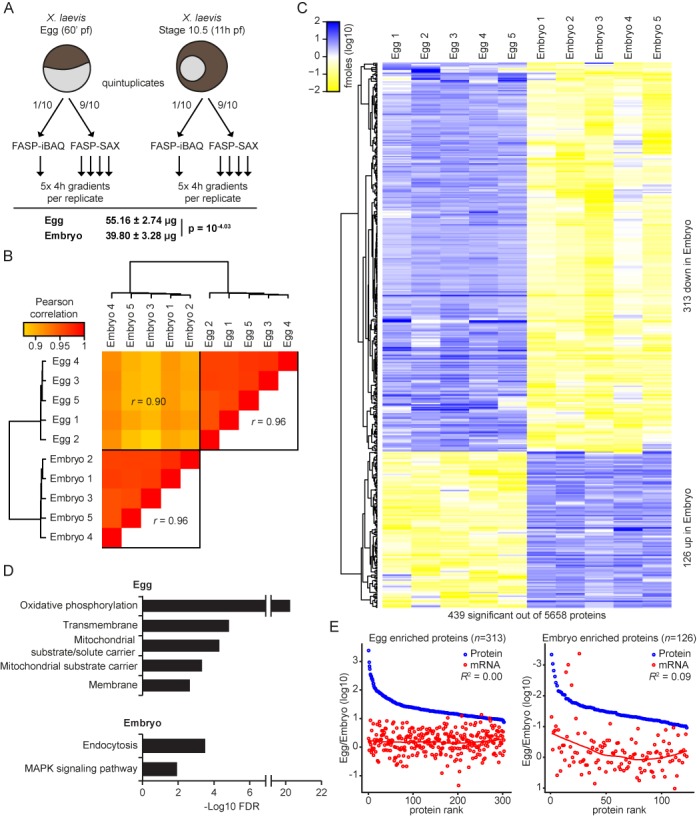
Dynamics of proteome and transcriptome in early *Xenopus* embryogenesis. (A) Schematic depiction of the workflow (upper panel) and the calculated protein content of *X. leavis* egg and embryo 10.5 (lower panel). (B) Unsupervised correlation-based clustering of egg and embryo 10.5 proteomes clearly separates egg and embryo 10.5 proteomes. (C) Heatmap of significant regulated proteins between egg and embryo 10.5, identified by an adapted *t*-test (FDR = 0.005 and s_0_ = 1). (D) Enriched GO terms for both egg and embryo 10.5 enriched proteins, as identified in C. *Y-*axis represents the –log FDR. (E) Protein and mRNA ratios (egg/embryo 10.5) for both egg (left panel) and embryo 10.5 (right panel) enriched proteins reveal lack of correlation between protein and mRNA dynamics. See also Supplementary Table S4.

### Mass spectrometry

Whole cell lysates were digested using filter-aided sample preparation (FASP) (30). For absolute quantification a standard range of proteins (UPS2, Sigma) was spiked into the sample (1:4 UPS2 to sample (μg/μg)) (31). For in-depth proteomics we applied the digested samples to strong anion exchange (SAX) (32), and we collected the flow through (FT) and pH11, pH8 and pH2 elutions. The peptides were subjected to Stage-Tip desalting and concentration (33) before mass spectrometry analysis. Samples were applied to online nanoLC-MS/MS, using 4 h gradients. For FASP samples, a 4–26% acetonitrile gradient followed by a step wise increase to 76% acetonitrile was used. For SAX samples, 4–14%, 6–17%, 7–18% and 9–21% acetonitrile gradients followed by a step wise increase to 76% acetonitrile were used for the FT, pH11, pH8 and pH2, respectively. Mass spectra were recorded on a LTQ-Orbitrap-Velos mass spectrometer (Thermo Scientific) using collision-induced dissociation (CID) fragmentation on the top 15 most intense precursor ions (data of Figure 1 and Supplementary Figure S1) or recorded on a Q Exactive mass spectrometer (Thermo Scientific) using higher-energy collisional dissociation (HCD) fragmentation on the top 10 most intense precursor ions (data of Figures 2–4).

### Database generation

We used the draft version of the *X. laevis* genome (JGI 7.1) produced by the International Xenopus laevis genome project consortium, downloaded from Xenbase (ftp://ftp.xenbase.org). We obtained gene annotation from several different sources (Supplementary Table S1). The Oktoberfest and Mayball gene annotation is produced by the International Xenopus laevis genome project. These sequences are described at the project website (http://www.marcottelab.org/index.php/Xenopus_Genome_Project). FASTA mRNA/EST/cDNA sequences were mapped to the *X. laevis* genome using gmap (26). All hits giving ≥90% identity were kept. Protein sequences were mapped to the *X. laevis* genome using blat (34). The blat alignments were processed using scipio (35), which corrects intron-exon borders and splice sites. In addition, transcript models predicted by Cufflinks (see RNA isolation and sequencing) were included. We called one optimal transcript per locus based on the following criteria: H3K4me3 ChIP-seq data at the 5′ end, level of RNA-seq expression (reads in exons as well as crossing splice junctions), length of the longest predicted open reading frame and number of different source including an exon. The database is available on the PRIDE repository (http://www.ebi.ac.uk/pride/archive/, PXD000902).

### Dimethyl labeling

After FASP, each sample was differentially labeled by incorporation of stable isotopes on the peptide level as described by Boersema *et al.* (36). Differentially dimethyl labeled samples were mixed, desalted and concentrated on Stage-Tips (33). Mixed labeled peptides were fractionated by SAX and measured on the mass spectrometer as described before, with the addition of an extra elution fraction at pH5.

### Proteomics data analysis

Raw mass spectrometry data were analyzed using MaxQuant (37), using default settings and with the algorithms ‘match between runs’ and ‘iBAQ’ enabled, and searched against the in-house generated database. The resulting identified proteins were filtered for contaminants and reverse hits. Proteins detected in the data from the unfractionated samples were filtered to be at least quantified in all nine triplicates (1670 proteins, Figure 1). Proteins detected in the quintuplicate egg analysis had to be quantified in all five replicates (5837 proteins, Figure 2) and for the comparison with mRNA levels the genes also needed to be quantified in all mRNA replicates (4612 proteins, Figure 3). For the combined egg and embryo analysis, proteins had to be quantified in either all replicates of the egg or all replicates of the embryo (5658 proteins, Figure 4). Missing values were semi-random imputed using Perseus (default settings, MaxQuant software package), based on the assumption that they were not detected because they were under or close to the detection limit. Unsupervised correlation-based clustering was performed in R. Identification of significant regulated proteins between egg and embryo 10.5 was done using an adapted *t*-test that is corrected for multiple testing by permutation-based estimation of the false discovery rate (FDR; default settings, Perseus of the MaxQuant software package). Gene ontology (GO) term enrichment were calculated with DAVID (38) for clusters of genes against the background of proteins identified and quantified in the corresponding sample. All proteomics data are available on the PRIDE repository (PXD000902).

## RESULTS

### Tight regulation of the proteome in single *X. laevis* eggs

To facilitate comprehensive mass spectrometry-based proteomics at the single-cell level, we made use of *X. laevis* eggs (60 min post-fertilization; 60’ pf) as a model system. A single egg contains enough material (roughly 50 μg non-yolk protein) to perform global absolute quantification of the proteome. We generated whole cell lysates of individual eggs and removed the yolk. In order to distinguish technical variation from biological noise, a cell lysate from a single egg was split into three and handled separately. This was done for three individual eggs from the same mother and all nine samples were prepared for mass spectrometry using FASP (Figure 1A) (30). To absolutely quantify all proteins, a standard range of proteins with a known molar concentration was spiked into each sample (UPS2, Sigma) and all measured intensities were normalized using the intensity-based absolute quantification algorithm (iBAQ) (31). The data were analyzed using MaxQuant and an in-house generated database (deposited at the PRIDE repository: PXD000902). Our database integrates data from various sources and outperforms the model system database in identification rates, identified peptides and quantified proteins (Supplementary Table S1). Note that 1670 proteins were identified in all nine replicates and quantified using linear regression (Figure 1, Supplementary Table S2). Strikingly, unsupervised clustering of the global proteomes revealed that technical and biological replicates could not be distinguished (Figure 1B). Furthermore, each of the nine experiments showed a high correlation (average *r* = 0.96). Altogether, this indicates that global absolute protein abundance in single *X. laevis* eggs is tightly regulated.

To achieve a greater depth, we set out to fractionate the egg proteome on the peptide level and measure these multiple fractions separately. To this end, we made use of SAX directly coupled after FASP-mediated protein digestion (Figure 2A; SAX sample) (32). A small aliquot (10%) of the whole cell lysate was combined with the UPS2 spike-in, which enabled absolute quantification (Figure 2A; iBAQ sample). The remainder of the lysate was subjected to FASP-SAX fractionation. Since we work with low quantities of protein, we optimized the SAX fractionation and eventually generated four different samples (FT, pH 11, 8 and 2). Furthermore, nano-HPLC acetonitrile gradients were also optimized for the low amounts of peptides present in the fractions (Supplementary Figure S1A). To assess the quality of FASP-SAX based quantification, we compared measurements on pools of eggs with measurements on individual eggs and found high correlations between technical and biological replicates (*r* = 0.96) (Supplementary Figure S1B). In contrast, a technical control comparing the proteome of HeLa and MCF7 cells using the same workflow revealed a significantly lower correlation (*r* = 0.81), whereas comparing two HeLa cell populations show correlations that are similar compared to those observed in single eggs (*r* = 0.98) (Supplementary Figure S1C). This indicates that FASP-SAX based peptide fractionation does not affect the quality of our quantifications. Moreover, the high correlation between the deep proteomes further substantiates the finding that the global proteome in individual eggs is tightly regulated. To further validate these findings, we also made use of a relative quantification method called dimethyl labeling (36). Relative quantification methods, which rely on stable isotope labeling, can identify smaller variations in abundance compared to absolute quantification methods. Dimethyl labeling-based quantification of biological and technical replicates revealed a similar number of outliers, indicating that biological noise is equal to or lower than the technical noise (Supplementary Figure S2). This further substantiates our previously described results based on absolute protein quantification, namely, that global protein abundance in single fertilized *X. laevis* eggs is tightly controlled.

Optimized FASP-SAX based fractionation allowed us to identify more than 5600 proteins in single eggs, and in total 5837 proteins in five biological replicates (Supplementary Table S3). Despite fractionation of the tryptic peptides and downscaling the amount of UPS2 standard, the absolute proteome of single eggs is of good quality (linear regression curve of the UPS2 standard = 0.96, Supplementary Table S2). This data set represents the largest single-cell proteome characterized to date. As shown in Figure 2B, the measured proteome covers more than seven orders of magnitude in abundance, of which five orders of magnitude were quantified with high accuracy. This indicates that the dynamic range of protein abundance in single *Xenopus* eggs is similar to that of cell populations in commonly studied cancer cell lines. Since we made use of the UPS2 spike-in standard, we were able to determine single cell copy numbers. Detected copy numbers range from hundreds of thousands to around ten trillion copies, the most abundant protein being actin. Until recently, copy number information in *X. laevis* eggs was restricted to a handful of proteins. We now provide accurate copy number information for thousands of proteins. The copy number values we deduced are often in very good to excellent agreement with published literature (Table 1 and Supplementary Table S4) (39–48).

**Table 1.** Protein copy numbers as determined in eggs (60’ pf, single cell) and embryos 10.5 (thousands of cells). Previously reported copy numbers for specific proteins are listed in the last column

	Number of molecules		
	Egg (60’ pf)	Embryo 10.5	Reported number
**Histones**			
Canonical histones	3.9 ± 1.0 * 10^12^	3.5 ± 0.7 * 10^12^	5 * 10^12^ (oocyte) (39)
H1B	1.3 ± 1.7 * 10^8^	1.2 ± 0.4 * 10^11^	-
H1foo	1.8 ± 0.5 * 10^11^	1.0 ± 0.4 * 10^11^	2.0 * 10^11^ (oocyte) (40)
**Chaperone**			
Nucleoplasmin 1,2,3	5.1 ± 0.7 * 10^12^	4.4 ± 0.4 * 10^12^	5 * 10^12^ (oocyte) (41)
**General transcription factors**			
gtf2b	2.2 ± 0.6 * 10^9^	2.2 ± 0.6 * 10^9^	-
gtf2e1	1.5 ± 3.0 * 10^7^	3.9 ± 1.8 * 10^8^	-
gtf2f1	1.4 ± 0.3 * 10^9^	1.7 ± 0.6 * 10^9^	-
taf9b	3.3 ± 0.8 * 10^8^	2.2 ± 0.7 * 10^8^	-
taf11	7.4 ± 5.3 * 10^7^	1.1 ± 0.4 * 10^8^	-
btaf1	3.3 ± 5.8 * 10^6^	1.5 ± 2.1 * 10^6^	-
**Transcription regulators**			
rest	5.1 ± 3.0 * 10^6^	2.9 ± 6.4 * 10^6^	-
tcf25	2.0 ± 1.0 * 10^8^	1.4 ± 0.6 * 10^8^	-
smad1/2/3/8/9	1.8 ± 0.5 * 10^9^	2.5 ± 0.6 * 10^9^	-
smad4.2	2.1 ± 0.6 * 10^8^	1.0 ± 0.5 * 10^8^	-
pou2f1 (oct1)	1.6 ± 3.4 * 10^9^	2.1 ± 1.9 * 10^7^	7.5 * 10^8^ (embryo) (42)
pou5f3.2 (oct25)	nd	4.0 ± 1.9 * 10^8^	-
tcf3	1.4 ± 0.8 * 10^7^	3.5 ± 1.0 * 10^8^	-
**Cytoplasmic deadenylation**			
EDEN-BP	1.0 ± 0.3 * 10^10^	3.0 ± 1.8 * 10^9^	-
**Cytoplasmic polyadenylation**			
Cpsf1	9.9 ± 3.9 * 10^8^	2.8 ± 1.2 * 10^8^	-
Cpeb1	1.7 ± 0.5 * 10^9^	8.0 ± 4.4 * 10^7^	-
Pumilio	1.9 ± 1.2 * 10^8^	2.7 ± 1.0 * 10^8^	-
**Housekeeping genes**			
gapdh	7.0 ± 1.4 * 10^12^	4.4 ± 0.6 * 10^12^	-
odc1	1.1 ± 2.5 * 10^6^	2.9 ± 1.1 * 10^7^	-
eef1a	3.8 ± 0.1 * 10^12^	2.3 ± 0.3 * 10^12^	-
**MAP Kinase**			
map2k1	1.9 ± 0.3 * 10^11^	1.9 ± 0.4 * 10^11^	2.4 * 10^11^ (oocyte) (43)
**Cell Cycle**			
cyclin a	2.9 ± 1.8 * 10^8^	1.2 ± 0.3 * 10^8^	3.6 * 10^9^ (oocyte) (44)
wasl	3.9 ± 0.7 * 10^8^	3.7 ± 1.1 * 10^8^	9.9 * 10^9^ (oocyte) (45)
**DNA replication**			
pcna	1.4 ± 0.1 * 10^12^	8.0 ± 2.0 * 10^11^	1.6 * 10^12^ (oocyte) (46)
topbp1	3.6 ± 2.6 * 10^8^	2.1 ± 1.4 * 10^7^	5.8 * 10^9^ (oocyte) (47)
geminin	1.0 ± 0.5 * 10^9^	5.2 ± 1.7 * 10^8^	2.0 * 10^9^ (oocyte) (48)

Based on their copy number, we divided all proteins into five bins (Figure 2C). The 374 most abundant proteins (6%) in single *Xenopus* eggs account for 90% of the total protein content. When visualizing the distribution of copy numbers, we observed a small shoulder on the right side of the plot, which contains proteins with the highest abundance. This cluster of proteins in strongly enriched for the translation machinery and metabolic enzymes. This overrepresentation of ribosomes and metabolic enzymes has not been observed in absolute proteomes of mammalian cell lines, whose copy number distribution shows a gamma distribution (31,49,50). These observations may underscore the unique biology of the egg and early embryo, which undergoes a large amount of cell divisions in a short period of time. Strikingly, Wnt signaling is enriched in a cluster of highly abundant proteins which also includes proteins involved in metabolism and replication. Less abundant proteins include transcription factors and proteins involved in ErbB signaling. Interestingly, although actin is the most abundant protein in the egg, some of the proteins involved in the cytoskeleton are present at low abundance.

### mRNA abundance is a poor predictor of protein abundance in *X. laevis* eggs

Recently published single-cell transcriptome analyses have shown heterogeneity of gene expression in mature mammalian cells. Transcript and protein levels in *X. laevis* embryos are known to be developmentally regulated by (post-)transcriptional and (post-)translational mechanisms. It is, however, not known to what extend this is a global effect. We observed a tight regulation of global absolute protein levels in single eggs. To investigate transcriptome heterogeneity in individual *Xenopus* eggs and to directly compare this to the single-cell proteome, we performed RNA-seq from five single eggs in parallel. These eggs were obtained from the same *in vitro* fertilization and originating from the same mother as the ones from which proteomes were generated, which enabled us to compare transcriptome and proteome from highly similar samples. The RNA samples were depleted from ribosomal RNA and sequenced on an Illumina HiSeq2000. Comparison of the average protein amounts and the average RNA-seq reads per kilobase per million reads (RPKM) showed a rather poor correlation (*R*^2^ = 0.17) (Figure 3A), very similar to the correlations at the single egg level (Supplementary Figure S3A). When only taking high-abundant proteins into account, which have been quantified within the detected UPS2 range (3574 proteins), the global mRNA—protein correlation remains similarly poor (Supplementary Figure S4A). Previous studies on global protein versus mRNA comparisons in mammalian cell populations have reported higher correlations (*R*^2^ = 0.40–0.50) (31,51). This difference may be caused by maternal loading of mRNA and protein in the eggs and translational regulation of maternal mRNAs. Interestingly, factors involved in deadenylation and polyadenylation which are known to regulate translation rates are highly abundant in *X. laevis* eggs and some of these are downregulated in embryos (Table 1). In any case, this low correlation highlights the importance of proteomic analysis as mRNA abundance is a poor predictor of protein abundance and function in *X. laevis* eggs. As was observed for the single-cell proteome, single-cell transcriptomes show a good correlation (*r* = 0.92) (Figure 3B). This correlation is similar compared to previously published single cell oocyte and zygote transcriptomes (52–54). Technical transcriptome replicates show a slightly higher correlation (*r* = 0.97) (Supplementary Figure S5C). Thus, even though the proteomes and transcriptomes are only moderately interrelated, in our measurements both single cell proteomes and transcriptomes are tightly controlled as they both show very little single cell variability.

The five individually measured proteomes and transcriptomes allowed us to determine the coefficient of variation (CV) for all mRNAs and proteins, which is a more sensitive way to examine variability. The CV for mRNA and protein range from 3% to 207% and 2% to 223%, respectively. As shown in Figure 3C, variation of proteins and mRNA on the single-cell level do not correlate. This indicates that noise in protein abundance is not caused by noise in mRNA abundance and therefore has to be caused by post-transcriptional and (post-)translational mechanisms.

We divided genes based on their mRNA and protein CV: genes with similar variation in mRNA and protein level (blue: low variation (1.9%) and green: high variation (2.1%)), genes with low mRNA variation and high protein variation (orange, 2.6%) and genes with high mRNA variation and low protein variation (red, 2.2%). Given the linear correlation between abundance and accuracy of the measurements, the highest abundant proteins and mRNA will be measured very accurate, whereas low abundant proteins and mRNA will have far more technical noise, therefore, the 20% highest and lowest abundant mRNA and proteins were omitted from this analysis (Supplementary Figure S3B). This analysis revealed that genes involved in endocytosis are tightly regulated at the protein level, whereas their mRNA abundance is noisier (Figure 4D). In contrast, genes involved in cell-cycle regulation and oocyte meiosis are tightly controlled at the transcriptome level, whereas protein abundance shows more variation. Interestingly, a group of sperm receptors (Zona pellucida glycoproteins: zp2, 3, 3.2 and 4 and zpax, zpd and ZPY1) show substantial variation at the protein level, whereas their mRNA is tightly regulated. These sperm receptors are degraded following sperm entry through the release of lysosome-like vesicles containing serine proteases. The high CV of these sperm receptors therefore may reflect a slightly different time point in sperm entry block between individual eggs. Finally, genes involved in splicing are tightly controlled at the transcript and protein level.

### Proteome changes during early *Xenopus* embryogenesis are only partly reflected by the transcriptome

To study the dynamics of gene expression at the transcript and protein level in *X. laevis*, we quantified the proteome and transcriptome in stage 10.5 embryos (zygotic activation of transcription is initiated at stage 8.5). At this stage, each embryo consists of thousands of cells. To profile the proteome, we again made use of the FASP-SAX workflow and iBAQ-based absolute quantification (Figure 4A). We identified and quantified a total of 5877 proteins in five embryos (Supplementary Table S4). Strikingly, the total amount of protein in embryos is lower compared to eggs (40 μg versus 55 μg, respectively). This may be due to protein degradation during early development, which apparently is more prominent than protein synthesis. The proteome in individual gastrula embryos is tightly controlled, similar to single eggs (average correlation *r* = 0.96; Figure 4B). This correlation remains strong when only taking high abundant proteins into account which have been quantified within the detected UPS2 range (3423 proteins) (Supplementary Figure S4B). In addition to the proteomes, transcriptomes were also obtained from stage 10.5 embryos. Both technical and biological transcriptome replicates show high correlations (*r* = 0.97 and 0.94, respectively), indicating tightly controlled transcriptomes (Supplementary Figure S5C). Interestingly, the protein and mRNA abundance correlation in stage 10.5 embryos is worse compared to the correlation in eggs (*R*^2^ = 0.09; Supplementary Figure S5A). mRNA and protein correlations in individual embryos are highly similar (Supplementary Figure S5B). The embryo and egg proteomes correlate less well (average correlation *r* = 0.90) (Figure 4B), thus revealing that part of the proteome is dynamic during early development.

To identify regulated proteins, *t*-test based statistics was applied to the data, which resulted in the identification of 439 significantly regulated proteins (7.8% of the complete proteome; Figure 4C, Supplementary Table S4). A total of 313 proteins were downregulated in embryos. This cluster of proteins is enriched for GO terms related to mitochondrial function (Figure 4D). Examples of downregulated proteins include ribosomal proteins (mrpl family of proteins), sperm receptors (zp 3.2 and 4), hells and ttf2. Note that 126 proteins were upregulated in embryos. The highest ranking embryo-enriched protein by *t*-value (Supplementary Table S4) is linker histone H1. This somatic linker histone is known to replace oocyte linker histone B4, which is associated with loss of mesoderm competence (55–57). Conversely, the oocyte linker histone is required for reprogramming of somatic cell nuclei to pluripotency (58). GO terms enriched in this cluster of genes are endocytosis and mitogen-activated protein kinase (MAPK) signaling. One of the upregulated proteins is Pou5f3, also called Oct25, an ortholog of the pluripotency transcription factor Oct4, which is involved in the activation of zygotic transcription in zebrafish (59,60). Perhaps also relevant in this context is the strong upregulation of the basal transcription factors TFIIE (two subunits) and TFIIA. Other basal transcription factors, such as TFIIF and TFIIB, are not regulated, as has been shown previously (61). Translation of maternal TBP mRNA is required for TBP protein accumulation in early development, and this seems a recurrent theme for transcription initiation factors based on the upregulation of TFIIE and TFIIA in stage 10.5 embryos (see Table 1).

Finally, we analyzed whether significant regulation at the protein level is caused by corresponding changes at the mRNA level. To this end, we compared the egg over embryo ratios for protein and mRNA in the eggs (Figure 4E, left figure) and the embryos (Figure 4E, right figure). This analysis revealed that protein dynamics do not correlate with changes at the mRNA level (*R*^2^ = 0.00 in eggs and *R*^2^ = 0.09 in embryos). To assess whether differential polyadenylation and deadenylation of transcripts may contribute to this discrepancy, the relative polyadenylation status of orthologous *Xenopus tropicalis* mRNAs in oocytes and early embryos (21) was compared for differentially regulated proteins. Proteins more abundant in eggs compared to early embryos showed relatively strong polyadenylation of the corresponding transcripts in oocytes and were subsequently deadenylated during early post-fertilization development (Supplementary Figure S6). The polyadenylation of proteins that are more abundant in embryos, however, showed less dramatic changes in polyadenylation. Altogether, these results imply that protein abundance changes might be regulated by translational rather than transcriptional mechanisms, which contributes to a sustained poor protein and transcript expression correlation during early *Xenopus* development. Therefore, mass spectrometry-based proteomics is an important tool to study protein expression dynamics during early embryogenesis.

## DISCUSSION

Here, we have presented the first absolute quantitative proteome of single *X. laevis* eggs to a depth of ∼5800 proteins, by far the largest single-cell proteome characterized to date. It is difficult to assess how comprehensive this proteome is. Of the 5800 proteins that we identified and quantified roughly 15% are covered by a single unique peptide. This indicates that we have not yet reached saturation in our data. Very recently, a very deep *Xenopus* egg proteome was published (62). The authors in this paper identified around 11.000 proteins, suggesting that our single-cell proteome covers 50–60% of the complete *Xenopus* egg proteome. Further technological improvements are needed to routinely characterize complete vertebrate proteomes from a limited amount (30–50 μg) of starting material and with minimal sample fractionation. Mammalian oocytes and zygotes are ∼10 times smaller (diameter) compared to *X. laevis* eggs. This suggests that a single mouse or human oocyte contains roughly 50 ng of protein, which is not yet sufficient to generate a comprehensive proteome. Comprehensive characterization of single somatic cells (which contain ∼0.1–0.5 ng of protein) using mass spectrometry-based proteomics is similarly not within reach in the foreseeable future.

Recently, Sun *et al.* reported a mass spectrometry-based screening of proteome dynamics during early *X. laevis* development (63). In this study, the authors identified and relatively quantified ∼4000 proteins using iTRAQ labeling. Our deeper absolute proteome covers more proteins involved in diverse cellular functions, such as basal transcription, metabolism, signaling and so forth (Supplementary Figure S7). We observed a good correlation (*R*^2^ = 0.68) between both studies with regards to significantly regulated proteins (60’pf versus stage 10.5/11) during early *X. laevis* embryogenesis. However, we identified some 200 significantly regulated proteins, which were not identified as developmentally regulated proteins in the Sun *et al.* publication, including interesting factors, such as Oct25 and TFIIE. Of note, the dynamic range of relative quantification is limited by the labeling efficiency (up to two orders of magnitude). In contrast, the dynamic range of developmentally regulated proteins in our absolute quantification is over six orders of magnitude.

One surprising observation we made is the fact that global protein abundance in single *X. laevis* eggs is tightly regulated, implying that there is a limited role for stochastic gene expression at the beginning of development. Having said this, the resolution of our measurements and the ability to detect significant differences is dependent on the abundance of each individual protein. High abundant proteins (top 10%) have a CV of ∼9%, whereas for lower abundant proteins (lowest 10%) this is on average 125%. In any case, we did not detect global significant heterogeneity in the single eggs that we characterized. Only a limited number of proteins seem to have heterogenic expression, which is somewhat in contrast to most of the published single-cell gene expression studies. However, recent transcriptome-based analysis of single mouse and human oocytes also reported a high correlation between individual cells (*r* = 0.90–0.98) (5,52–54). We observed similar single-cell transcriptome correlations in *X. laevis* eggs (*r* = 0.92). In contrast, single cell transcriptomes in somatic cells and cancer cell lines correlate less well (*r* = 0.54 – 0.83) (64,65). Several explanations could account for this difference between oocytes/embryos and single mammalian cells. First of all, oocytes or zygotes all represent a defined stage of development, have the same age and proceed in a synchronized manner through early cell divisions. Single somatic cells can all be in a different developmental stage, can be in a different phase of the cell cycle or may have acquired genetic alterations. Second, the limited amount of RNA in relatively small single somatic cells might result in noisier single-cell transcriptome data, either due to technical limitations or due to the impact of transcriptional bursting on the (far smaller) total pool of mRNA. Finally, the first single cell gene expression studies have all been done in unicellular organisms, such as *E. coli* and *S. cerevisiae* (6–8,10). Plasticity at the single-cell level caused by stochastic gene expression may also be more relevant for these species to cope with environmental stress.

Meiotic maturation and fertilization are associated with waves of deadenylation and polyadenylation of subsets of transcripts, influencing their stability and translation (21,66,67). Strikingly, the correlation between mRNA and protein decreases in stage 10.5 embryos. Maternal mRNA and protein are still dominating the transcriptome and proteome at this stage, and zygotic transcription has just started 5 h prior to stage 10.5. The combination of these parameters and mRNA and protein degradation apparently result in a decrease of correlation between global mRNA and protein abundance. Furthermore, the variation detected in protein levels between eggs is not caused by noise of corresponding mRNA. This suggests that post-transcriptional regulation, translation and protein degradation are the major constituents causing protein variation. Finally, we observe that developmental proteome dynamics are not mirrored by mRNA changes, implying that the majority of developmental changes in protein levels are disconnected from mRNA levels. Ultimately, our integration of proteomics and transcriptomics on single cells reveals that early *Xenopus* embryogenesis is characterized by a major decoupling of mRNA and protein levels with relatively little variability in both protein and RNA levels. Technological developments that will make it possible to perform comprehensive single cell proteomics on smaller cells, such as mammalian oocytes and even somatic cells, are essential to determine whether our findings can be extended to mammalian eggs and early embryos which have a much lower maternal contribution of mRNA and protein. Moreover, proteomic analyses of individual blastomeres will uncover how gene expression variability relates to cell size and developmental progression.

## ACCESSION NUMBERS

RNA sequencing data have been deposited to the Gene Expression Omnibus under accession number GSE56586. The mass spectrometry proteomics data and the in-house generated database have been deposited to the ProteomeXchange Consortium (http://proteomecentral.proteomexchange.org) via the PRIDE partner repository (68) with the data set identifier PXD000902.

## SUPPLEMENTARY DATA

Supplementary Data are available at NAR Online.

SUPPLEMENTARY DATA
