# Supplementary material for: Global absolute quantification reveals tight regulation of protein expression in single Xenopus eggs
Source: Nucleic Acids Res. 2014 Jul 23;42(15):9880–91. doi: 10.1093/nar/gku661 (PMC4150773; doi:10.1093/nar/gku661)
Supplement: SUPPLEMENTARY DATA [file supp_42_15_9880__index.html]

Global absolute quantification reveals tight regulation of protein expression in single Xenopus eggs — Global absolute quantification reveals tight regulation of protein expression in single Xenopus eggs — SUPPLEMENTARY DATA 

# Global absolute quantification reveals tight regulation of protein expression in single *Xenopus* eggs

## SUPPLEMENTARY DATA

**Files in this Data Supplement:**

- SUPPLEMENTARY DATA
- SUPPLEMENTARY DATA
- SUPPLEMENTARY DATA
- SUPPLEMENTARY DATA
- SUPPLEMENTARY DATA
