# Supplementary material for: Global absolute quantification reveals tight regulation of protein expression in single Xenopus eggs
Source: Nucleic Acids Res. 2014 Jul 23;42(15):9880–91. doi: 10.1093/nar/gku661 (PMC4150773; doi:10.1093/nar/gku661)
Supplement: SUPPLEMENTARY DATA [file supp_gku661_nar-01175-h-2014-File007.pdf]

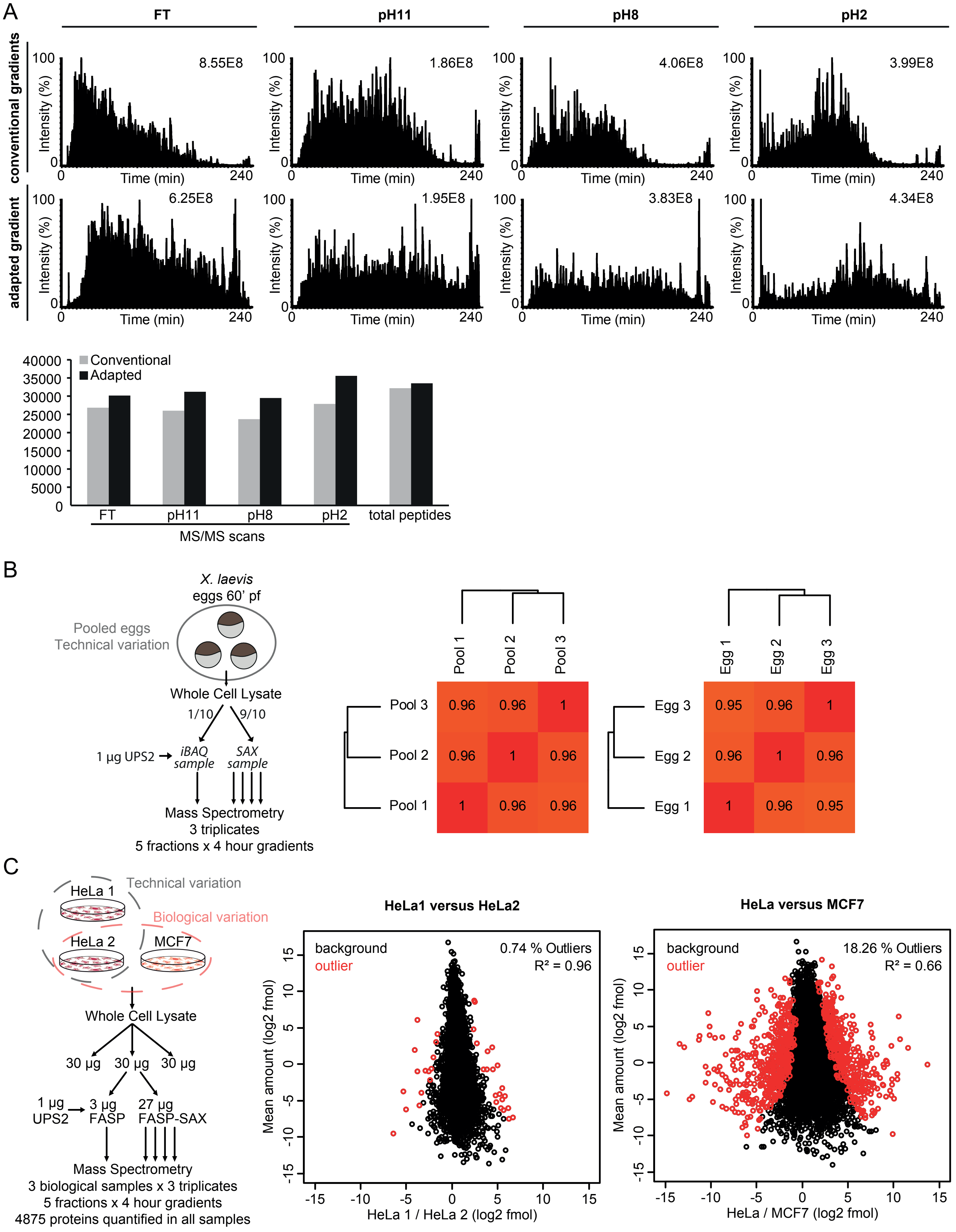

Figure S1, Optimization of nanoLC gradients and quality control of absolute quantification in the fractionated samples, related to Figure 2. (A) Conventional and optimized nanoLC gradients for the four different fractions (upper panel) and the number of informative MS/MS scans as well as the total number of identified peptides for these gradients (lower panel). Four fractions (FT, pH11, pH8 and pH2) resulted in the most extensive fractionation with high identification rates. **(B)** Quality control of absolute quantification in the fractionated samples from technical replicates from pooled *X. leavis* eggs and from single *X. leavis* reveal tight correlation of in-depth egg proteomes. **(C)** Quality control of absolute quantification of fractionated samples from HeLa and MCF7 lysates reveal a high technical reproducibility (HeLa1 versus HeLa2) and high sensitivity in calling differential expressed proteins (HeLa vs MCF7). The significance threshold is calculated using an binned intensity-corrected 1% FDR (50 intensity bins, y-axis).

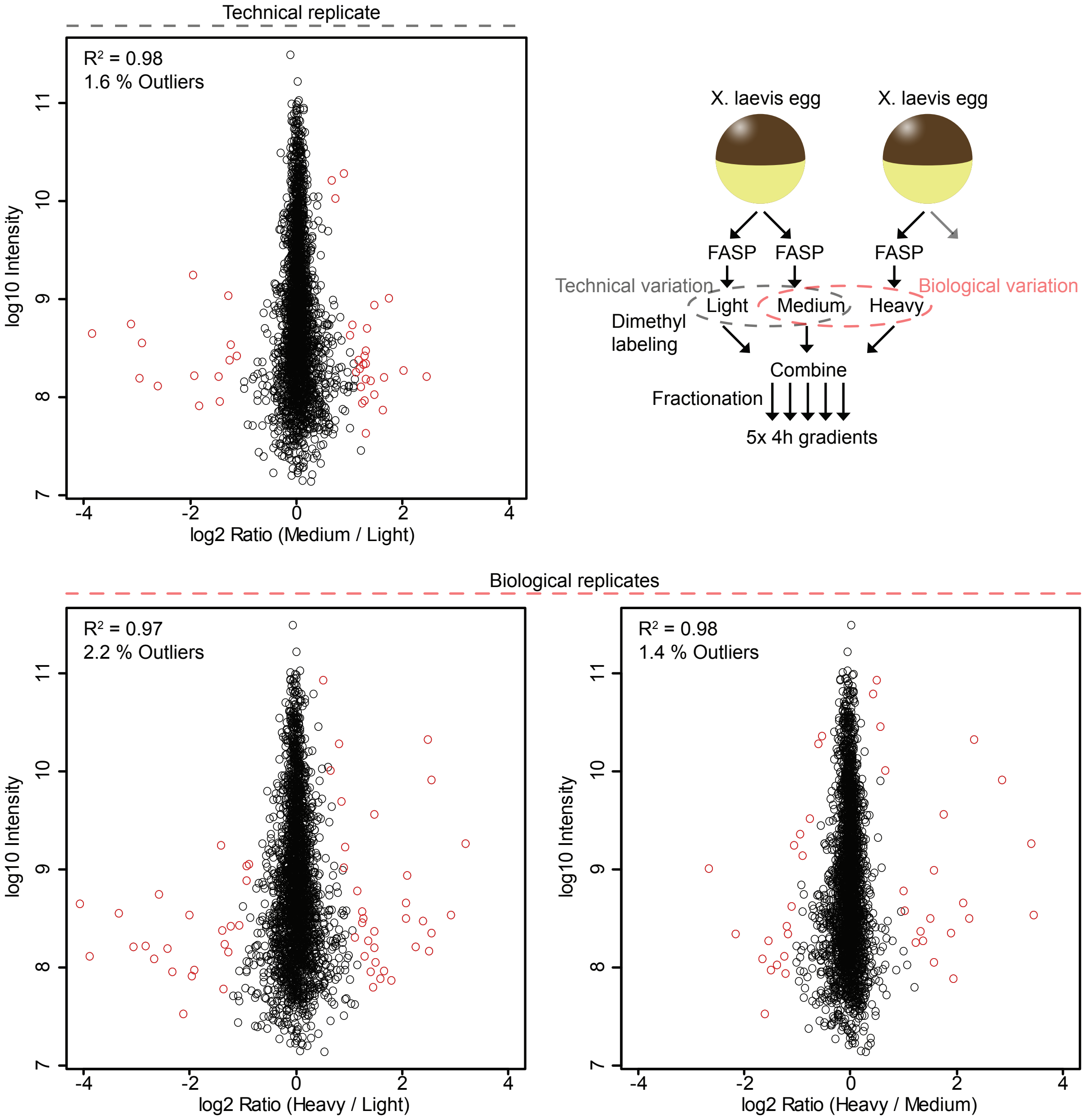

Figure S2, relative quantitation of single cell proteomes in Xenopus laevis eggs using dimethyl labeling. An overview of the workflow used to detect technical and biological variation in single cell proteomics is presented on the right. The scatterplot for the technical and biological comparisons are depicted, with the ratios on the x-axis and the measured intensities on the y-axis. The significance threshold is calculated using an binned intensity-corrected 1% FDR (50 intensity bins, y-axis).

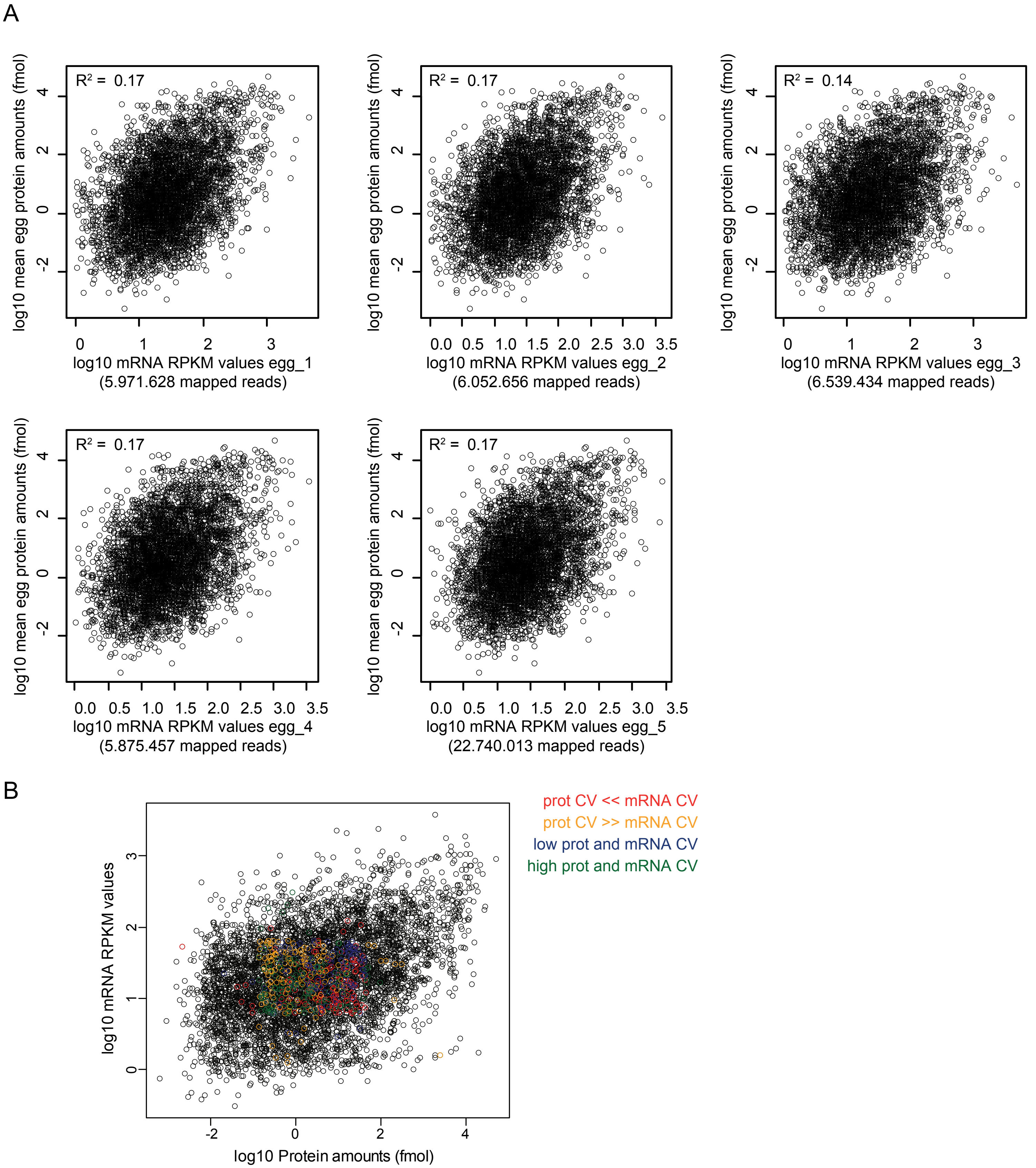

Figure S3, protein and mRNA abundance correlations of individual eggs, related to Figure 3. (A) Scatterplots of mRNA levels measured in single eggs against the mean egg protein abundance reveal highly similar protein-mRNA correlations in single cells. **(B)** Scatterplot of the mean mRNA versus the mean protein abundance in which the proteins are depicted from the different protein clusters in Figure 3C.

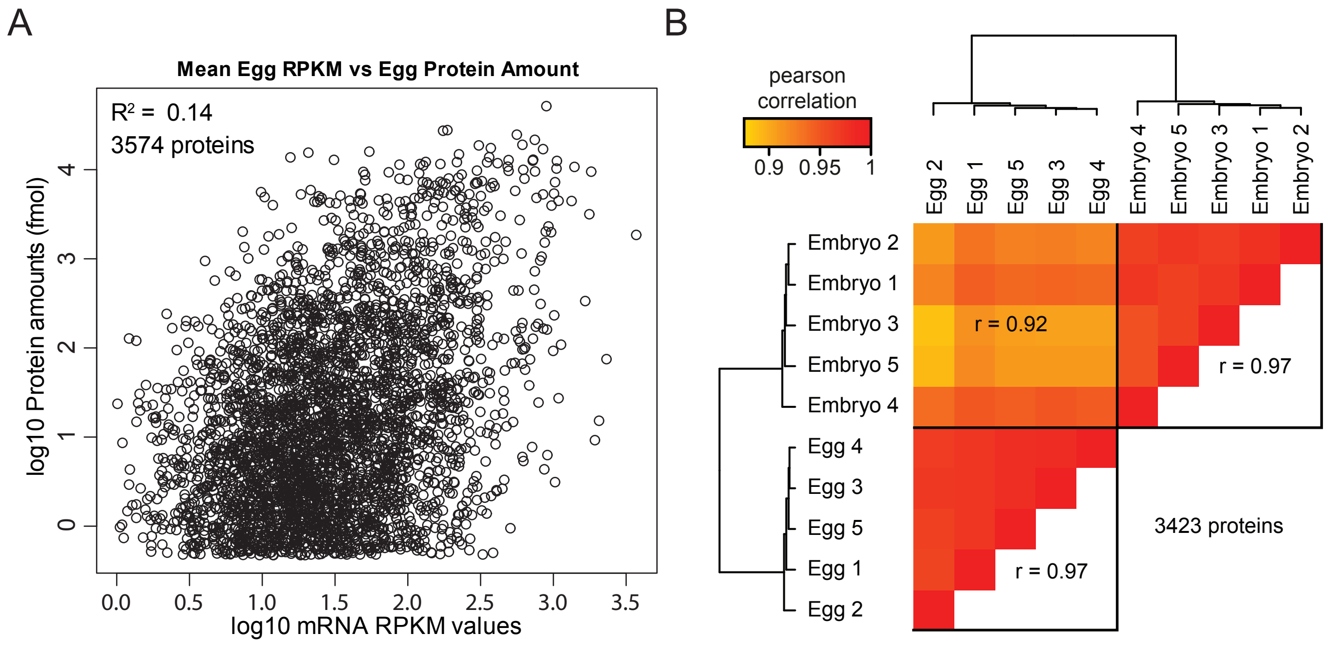

Figure S4, analysis of a subset of the data containing high abundant proteins (detected UPS2 range) shows highly similar correlations compared with correlations found using the whole dataset **(A)** Scatterplot of the proteome and transcriptome in the Xenopus egg. **(B)** Correlations between the whole proteomes of single eggs and embryos.

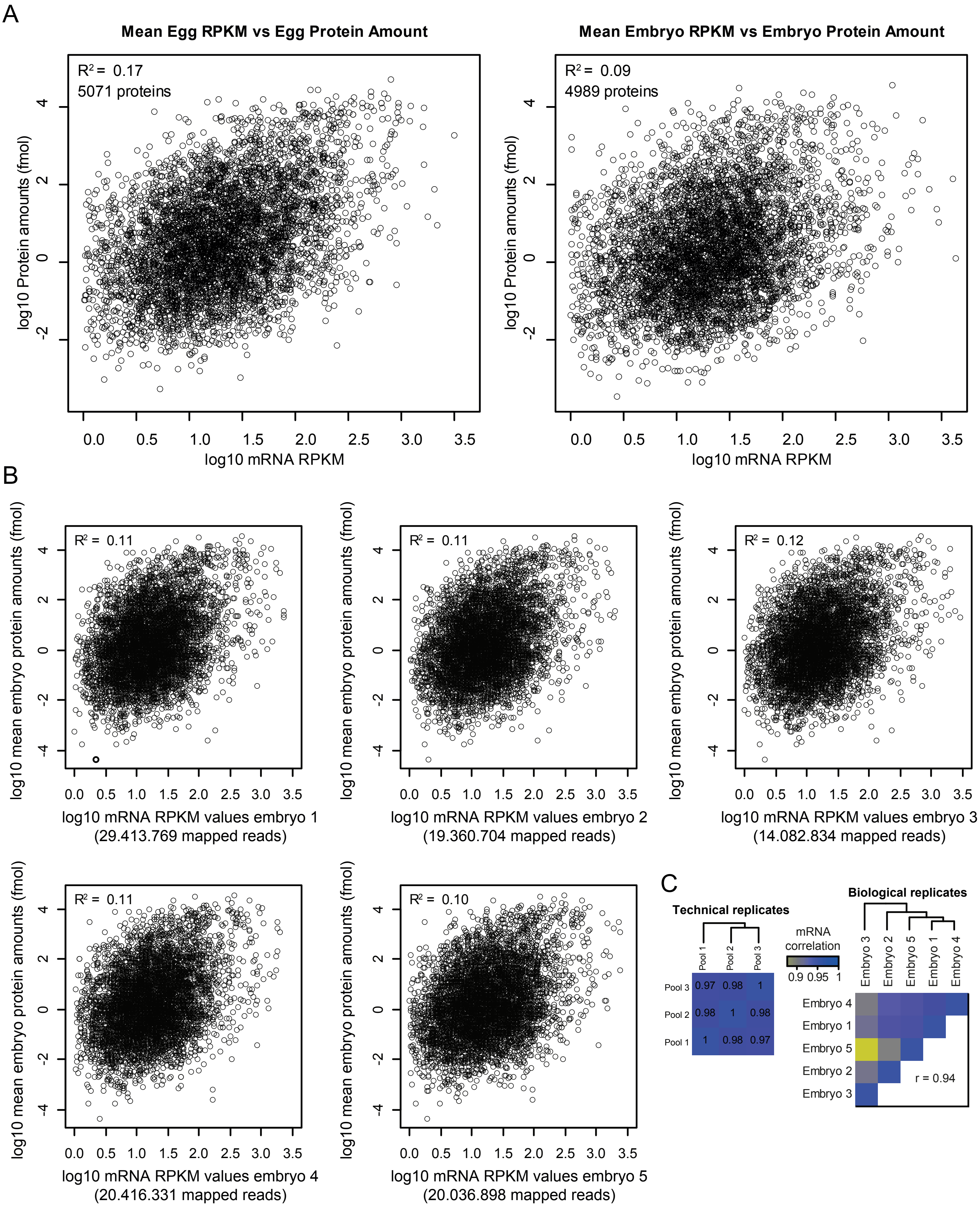

Figure S5, protein and mRNA abundance correlations of individual embryos (stage 10.5), related to Figure 4. (A) Scatterplots of mean mRNA versus mean protein abundance for both egg (left) and embryo 10.5 (right) using the catenated RNA-Seq profiles. This analysis reveals that the mRNA-protein abundance correlation is decreasing during the early Xenopus embryogenesis. **(B)** Scatterplots of mRNA levels measured in single embryos against the mean embryo protein abundance reveal that the correlations vary on single cell level, which is probably due to lower amounts of mapped reads. **(C)** Correlations between technical and biological transcriptome replicates.

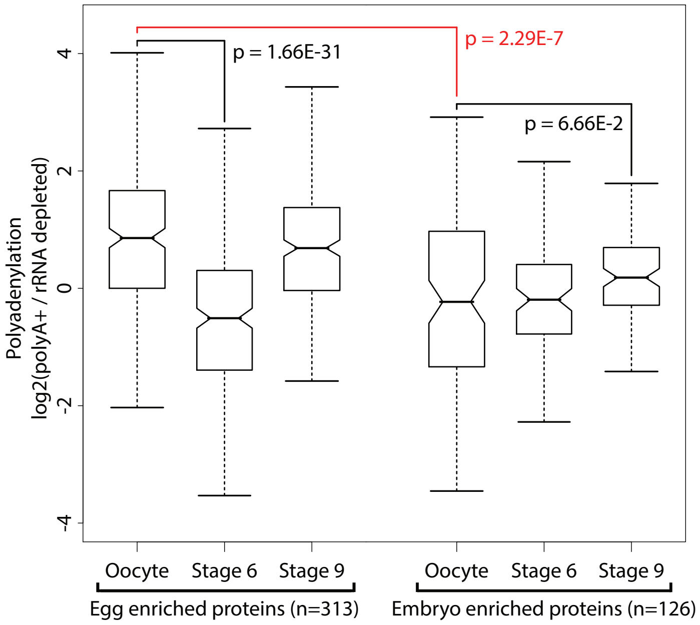

Figure S6, polyadenylation state for the corresponding transcripts of significant regulated proteins between egg and embryo. Boxplots of estimated polyadenylation state (polyA enrichment over rRNA depleted ratio) for corresponding transcripts of proteins that are significantly more abundant in eggs (first three boxplots) and embryos (last three boxplots).

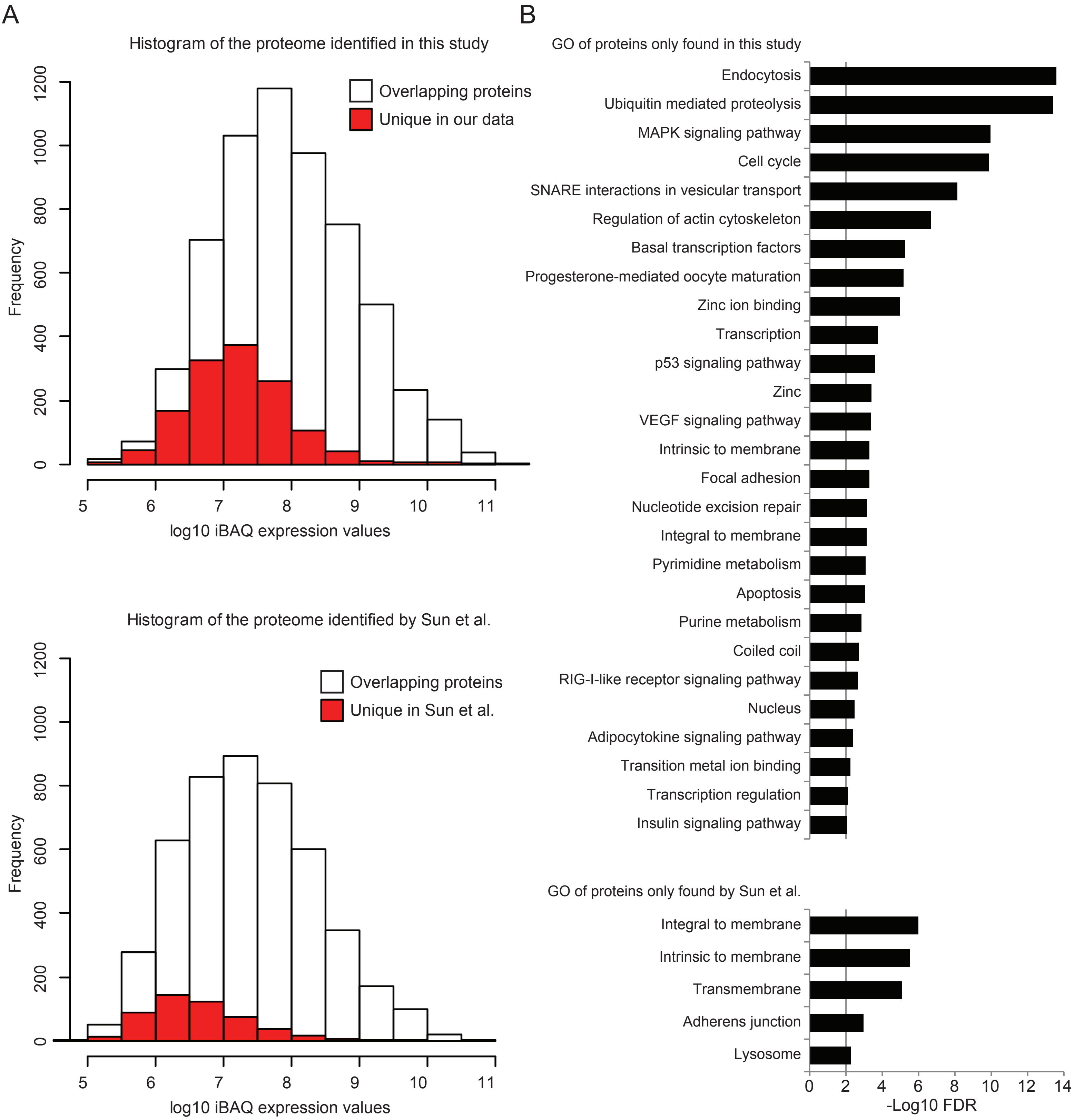

Figure S7, comparison of our data with Sun et al. (A) Histograms of our proteome (upper panel) and the proteome identified by Sun et al (lower panel). Proteins uniquely identified in our proteome or the proteome by Sun et al are in red. **(B)** Enriched GO terms for those unique proteins.
